# The relationship between childhood trauma, dopamine release and dexamphetamine-induced positive psychotic symptoms: a [^11^C]-(+)-PHNO PET study

**DOI:** 10.1038/s41398-019-0627-y

**Published:** 2019-11-11

**Authors:** Tarik Dahoun, Matthew M. Nour, Robert A. McCutcheon, Rick A. Adams, Michael A. P. Bloomfield, Oliver D. Howes

**Affiliations:** 1 Psychiatric Imaging Group, Robert Steiner MRI Unit, MRC London Institute of Medical Sciences, Hammersmith Hospital, London, W12 0NN UK; 2 Institute of Clinical Sciences, Faculty of Medicine, Imperial College London, Hammersmith Hospital, London, W12 0NN UK; 3 Department of Psychiatry, University of Oxford, Warneford Hospital, Oxford, OX37 JX UK; 4 Department of Psychosis Studies, Institute of Psychiatry, Psychology & Neuroscience (IoPPN), King’s College London, London, SE5 8AF UK; 5 Max Planck UCL Centre for Computational Psychiatry and Ageing Research, University College London, Russell Square House, 10-12 Russell Square, London, WC1B 5EH UK; 6 Wellcome Centre for Human Neuroimaging (WCHN), University College London, London, WC1N 3AR UK; 7 Division of Psychiatry, University College London, 6th Floor, Maple House, 149 Tottenham Court Road, London, WC1T 7NF UK; 8 Institute of Cognitive Neuroscience, University College London, 17 Queen Square, London, WC1N 3AZ UK; 9 Clinical Psychopharmacology Unit, Research Department of Clinical, Educational and Health Psychology, University College London, 1-19 Torrington Place, London, WC1E 6BT UK; 10 NIHR University College London Hospitals Biomedical Research Centre, University College Hospital, London, W1T 7DN UK; 11 Translational Psychiatry Research Group, Research Department of Mental Health Neuroscience, Division of Psychiatry, University College London, 6th Floor, Maple House, 149 Tottenham Court Road, London, WC1T 7NF UK

**Keywords:** Neuroscience, Psychiatric disorders

## Abstract

Childhood trauma is a risk factor for psychosis. Amphetamine increases synaptic striatal dopamine levels and can induce positive psychotic symptoms in healthy individuals and patients with schizophrenia. Socio-developmental hypotheses of psychosis propose that childhood trauma and other environmental risk factors sensitize the dopamine system to increase the risk of psychotic symptoms, but this remains to be tested in humans. We used [^11^C]-(+)-PHNO positron emission tomography to measure striatal dopamine-2/3 receptor (D_2/3_R) availability and ventral striatal dexamphetamine-induced dopamine release in healthy participants (*n* = 24). The relationships between dexamphetamine-induced dopamine release, dexamphetamine-induced positive psychotic symptoms using the Positive and Negative Syndrome Scale (PANSS), and childhood trauma using the Childhood Trauma Questionnaire (CTQ) were assessed using linear regression and mediation analyses, with childhood trauma as the independent variable, dexamphetamine-induced dopamine release as the mediator variable, and dexamphetamine-induced symptoms as the dependent variable. There was a significant interaction between childhood trauma and ventral striatal dopamine release in predicting dexamphetamine-induced positive psychotic symptoms (standardized *β* = 1.83, *p* = 0.003), but a mediation analysis was not significant (standardized *β* = −0.18, *p* = 0.158). There were no significant effects of dopamine release and childhood trauma on change in negative (*p* = 0.280) or general PANSS symptoms (*p* = 0.061), and there was no relationship between ventral striatal baseline D_2/3_R availability and positive symptoms (*p* = 0.368). This indicates childhood trauma and dopamine release interact to influence the induction of positive psychotic symptoms. This is not consistent with a simple sensitization hypothesis, but suggests that childhood trauma moderates the cognitive response to dopamine release to make psychotic experiences more likely.

## Introduction

Schizophrenia is among the leading causes of global disease burden and is associated with high mortality and morbidity^1–3^. It is a complex disorder with a multi-factorial aetiology involving both neurobiological alterations and environmental risk factors^4,5^. However, the relationship between neurobiological and environmental risk factors, and how they interact to increase the risk of schizophrenia and other psychotic disorders, is currently not known.

The dopamine hypothesis has been a leading neurobiological theory of schizophrenia for several decades^6–11^. Supporting the theory, administration of drugs that increase dopamine levels such as amphetamine or cocaine induces psychotic symptoms in both healthy people^12–14^ and patients with schizophrenia^15^. Molecular imaging studies have characterized the nature of the in vivo dopaminergic changes in schizophrenia, showing higher dopamine synthesis capacity and amphetamine-induced dopamine release with large effect sizes in patients with the disorder^6^. To our knowledge, seven of eight imaging studies found that schizophrenia is associated with increased striatal dopamine release induced by amphetamine^16–23^, and a further study found greater striatal dopamine release induced by acute stress exposure^24^. In addition, a number of these studies report a direct correlation between the magnitude of amphetamine-induced dopamine release in the striatum and amphetamine-induced psychotic symptoms in patients with psychosis^16–18^. However, individuals vary in the degree to which amphetamine induces psychotic symptoms^14^, suggesting that other factors may moderate individual vulnerability.

One of the most consistent environmental risk factors associated with schizophrenia is experiencing trauma in childhood and adolescence. Childhood traumas, such as neglect, physical, sexual and psychological abuse, increase the risk for psychosis in adulthood with odds ratios of 2.8–3.6 (refs. ^25–28^). Large population-based studies also found positive dose–response relationships between trauma and subclinical psychotic symptoms as well as the persistence of psychotic experiences in the general population^29–34^, suggesting that childhood trauma may increase the risk for developing psychotic symptoms as well as a psychotic disorder. However, not everyone exposed to childhood trauma develops psychosis, suggesting biological vulnerability is also relevant.

It has been proposed that childhood trauma, and other environmental stressors, sensitizes the mesostriatal dopamine system to the effects of later challenges, such as amphetamine, subsequently leading to the development of psychotic symptoms^4,35–38^. Supporting this, preclinical studies in rodents show increased amphetamine-induced dopamine release in the ventral striatum in animals exposed to a range of stressors during early development, including maternal deprivation^39^, neonatal isolation^40,41^, social isolation and social defeat^42^. Moreover, in vivo human positron emission tomography (PET) studies show that humans who reported lower maternal care during childhood have greater stress-induced dopamine release^43^, and participants who have experienced a greater number of traumatic events in childhood and higher levels of perceived stress have elevated levels of amphetamine-induced dopamine release^44^, both in the ventral striatum. In addition, increased dopamine synthesis capacity has been found in adults exposed to childhood adversity (both healthy individuals and those at ultra-high risk of psychosis)^45^. However, to our knowledge, the relationships between childhood trauma, amphetamine-induced dopamine release and induced psychotic symptoms remain poorly understood. In particular, it remains unknown whether childhood trauma and amphetamine-induced dopamine release are associated with induced positive psychotic symptoms in isolation, whether childhood trauma interacts with amphetamine-induced dopamine release to induce positive psychotic symptoms (moderation effect) or whether childhood trauma influences induced psychotic symptoms through amphetamine-induced dopamine release (mediation effect).

In view of this, we tested the relationships between childhood trauma and dexamphetamine-induced striatal dopamine release to predict dexamphetamine-induced positive psychotic symptoms using both moderation and mediation models. We also tested whether these relationships were specific to striatal dopamine release capacity, or whether they were also related to baseline striatal dopamine-2/3 receptor (D_2/3_R) availability. We focused on the ventral striatum as this region has previously been shown to be sensitive to the effect of childhood adversity on the magnitude of amphetamine-induced dopamine release^43,44,46^. We hypothesized that the interaction and/or mediation between childhood trauma and dopamine release capacity would predict dexamphetamine-induced positive psychotic symptoms, in light of the sensitization of the mesostriatal dopamine system described above^4,35–38^, and evidence showing that dopamine release in isolation is not correlated with an increase in amphetamine-induced positive symptom scores in young adults with hearing impairment^47^.

## Materials and methods

### Participants

The study was approved by the West London & GTAC NHS research ethics committee (12/LO/1955). Participants were recruited via online and newspaper advertisements. All participants gave informed written consent to take part in the study after its full description. The inclusion criteria for the study were (1) age above 18 years and (2) capacity to give written informed consent. The exclusion criteria were (1) any past or current major medical condition; (2) history of a psychiatric disorder as determined by the Structured Clinical Interview for DSM-IV Axis 1 Disorders, Clinician Version (SCID-CV)^48^; (3) history of substance abuse/dependence as determined by the SCID-CV; (4) history of head injury with a loss of consciousness; (5) a family history of any psychiatric disorder in first- or second-degree relatives; (6) contraindications to PET scanning (significant prior exposure to radiation, pregnancy or breast feeding); (7) positive urine drug screen, including for cannabis^49,50^.

### Overall description of the study

**Fig. 1:**
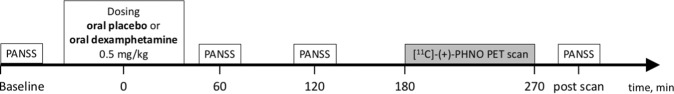
Timeline of scanning day. Time is represented on the *x*-axis and is relative to the administration of placebo or dexamphetamine. Participants underwent two [^11^C]-(+)-PHNO PET scans on separate days: (1) one following a single oral dose of placebo; (2) one following a single oral dose of dexamphetamine (500 µg/kg). Participants completed the Childhood Trauma Questionnaire on the first scan day. PANSS: Positive and Negative Syndrome Scale; [^11^C]-(+)-PHNO PET scan: [^11^C]-(+)-4-propyl-9-hydroxynaphthoxazin positron emission tomography scan

The timeline of scanning day is shown in Fig. 1. Participants attended a screening visit to check eligibility criteria, and underwent two [^11^C]-(+)-4-propyl-9-hydroxynaphthoxazin (PHNO) PET scans on separate days: the first following a single oral dose of placebo, and the second following a single oral dose of dexamphetamine (500 µg/kg) in a fixed order. On the first scan day, participants completed the Childhood Trauma Questionnaire (CTQ). On both scan days, positive (psychotic) symptoms, negative symptoms and general psychopathology symptoms were assessed using the Positive and Negative Syndrome Scale (PANSS) at baseline (pre-dosing with placebo or dexamphetamine), 60 min post-dosing, 120 min post-dosing, and after the scan (275 min post-dosing). Participants were blind to placebo/dexamphetamine administration. The rater was blind to childhood trauma load and dexamphetamine-induced dopamine release. All participants provided urine samples prior to each scan to screen for current recreational drug use. Participants were asked to abstain from smoking, drinking coffee, eating and using alcohol overnight (minimum 10 h) prior to their PET scans.

### Assessment of childhood trauma

Childhood trauma was assessed with the self-completed CTQ 25-item short form^51,52^, a reliable and valid measure of childhood trauma in patients with psychosis^53^. The CTQ is a retrospective self-completed questionnaire covering the following five domains of childhood trauma: sexual abuse, physical abuse, emotional abuse, physical neglect and emotional neglect^51,52^. Sexual abuse is defined as “sexual contact or conduct between a child younger than 18 years of age and an adult or older person”. Physical abuse is defined as “bodily assaults on a child by an adult or older person that posed a risk of or resulted in injury”. Emotional abuse is defined as “verbal assaults on a child’s sense of worth or well-being or any humiliating or demeaning behaviour directed toward a child by an adult or older person”. Physical neglect is defined as “the failure of caretakers to provide for a child’s basic physical needs, including food, shelter, clothing, safety, and health care”. Emotional neglect is defined as “the failure of caretakers to meet children’s basic emotional and psychological needs, including love, belonging, nurturance, and support”. In the short version of the CTQ, each domain includes five items (e.g. for the physical abuse subscale: (1) “I got hit so hard by someone in my family that I had to see a doctor or go to the hospital”; (2) “I was punished with a belt, a board, a cord, or some other hard object”; (3) “I believe that I was physically abused”; (4) “I got hit or beaten so badly that it was noticed by someone like a teacher, neighbor, or doctor”; (5) “People in my family hit me so hard that it left me with bruises or marks”). Participants have to rate the frequency to which they have been exposed to each item (“never”, “rarely”, “sometimes”, “often” or “very often”, total minimum CTQ score = 25, total maximum CTQ score = 125). For each participant, the sum of all the 25 items in the CTQ was used in the analyses.

### Assessment of dexamphetamine-induced psychotic symptoms

Positive, negative and general psychopathology symptoms were assessed during both placebo and dexamphetamine scans using the PANSS. The PANSS is an established clinician-rated scale used for measuring the severity of psychotic and related symptoms based on a 30-item standardized interview^54^. Each of the 30 items is accompanied by a specific definition as well as detailed anchoring criteria for all seven rating points. These seven points represent increasing levels of psychopathology from 1 (absent) to 7 (extreme)^54^. The symptoms assessed for the positive dimension are delusions, conceptual disorganization, hallucinatory behaviour, excitement, grandiosity, suspiciousness/persecution and hostility. The symptoms assessed for the negative dimension are blunted affect, emotional withdrawal, poor rapport, passive/apathetic social withdrawal, difficulty in abstract thinking, lack of spontaneity & flow of conversation, stereotyped thinking. The symptoms assessed for the general dimension are somatic concern, anxiety, guilt feelings, tension, mannerisms & posturing, depression, motor retardation, uncooperativeness, unusual thought content, disorientation, poor attention, lack of judgement & insight, disturbance of volition, poor impulse control, preoccupation and active social avoidance. For each participant, the sums of the seven sub-items for positive symptoms, seven sub-items for negative symptoms and 16 sub-items for general symptoms were calculated and used in the analyses for the positive, negative and general symptoms, respectively.

### Data acquisition

#### [^11 ^C]-PHNO PET data acquisition

PET images were acquired using a Siemens Biograph HiRez XVI PET scanner (Siemens Healthcare, Erlangen, Germany). A low-dose computed tomography scan was first obtained for attenuation and model-based scatter correction followed by the injection of a single intravenous bolus of 0.020–0.029 µg/kg [^11^C]-(+)-PHNO. All participants (*n* = 24) had a baseline PET scan and a PET scan following administration of dexamphetamine on a separate day in a fixed order. For the dexamphetamine PET scans, 500 µg/kg dexamphetamine was administered orally 3 h before [^11^C]-(+)-PHNO administration, so that the expected time of peak drug levels coincided with the scan time^55^. Previous studies have shown robust changes in dexamphetamine-induced dopamine release using this approach^22,56^. Dynamic emission data were acquired continuously for 90 min after the administration of the radiotracer. The dynamic images were then reconstructed using a filtered back-projection algorithm into 31 frames (8 × 15 s, 3 × 60 s, 5 × 120 s, 15 × 300 s) with a 128 matrix, a zoom of 2.6 and a transaxial Gaussian filter of 5 mm.

#### Structural MRI acquisition

The PET spatial pre-processing pipeline required a high-resolution structural magnetic resonance imaging (MRI) scan for each subject. MR images were acquired on a Siemens MAGNETOM Verio 3T MRI scanner and a 32-channel phased-array head-coil. A high-resolution T1-weighted volume was acquired for PET coregistration using a Magnetization Prepared Rapid Gradient Echo (MPRAGE) sequence with parameters from the Alzheimer’s Disease Research Network (ADNI-GO; 160 slices × 240 × 256, TR = 2300 ms, TE = 2.98 ms, flip angle = 9°, 1 mm isotropic voxels, bandwidth = 240 Hz/pixel, parallel imaging (PI) factor = 2)^57^.

#### PET analysis

PET images were analysed using MATLAB version 2015B and an automatic analysis pipeline implemented in MIAKAT (MIAKAT release 4.2.6, www.miakat.org)^58^. The ICBM152 high-resolution structural MRI template in Montréal Neurologic Institute (MNI) space was non-linearly warped to the high-resolution T_1_-weighted MRI of each participant using Statistical Parametric Mapping (SPM) (Wellcome Trust Centre for Neuroimaging). The derived deformation parameters were then applied to the Martinez striatal atlas, which defines the anatomical extents of the limbic (ventral), associative and sensorimotor striatal regions of interest (ROIs) in MNI space^59,60^, and the atlas used by Tziortzi et al. to define a cerebellar ROI to be used as the reference region^61^. The application of deformation parameters brings the ROIs into the native space of each subject’s MRI scan. The MRI and ROIs were then downsampled to the PET resolution (2 mm). A frame-by-frame registration process on a single frame of reference was used for motion correction for dynamic PET images. Individual averaged PET images were then co-registered to their respective MRIs using rigid body co-registration.

Regional time activity curves (TACs) were obtained by applying individual parcellations to the realigned dynamic images. Our outcome measure of interest was non-displaceable binding (BP_ND_) of [^11^C]-(+)-PHNO:

$${\mathrm {BP}}_{{\mathrm {ND}}} = \frac{{f_{{\mathrm {ND}}}B_{{\mathrm {avail}}}}}{{K_{\mathrm {D}}}},$$

where *B*_avail_ is the proportion of D_2/3_Rs available to be bound by PHNO (i.e. the fraction of receptors not bound by endogenous synaptic dopamine), *f*_ND_ is the free fraction of PHNO in the brain and 1/*K*_D_ the affinity of ligand for the target. BP_ND_ was obtained by kinetic modelling with a simplified reference tissue model^62,63^, using the whole cerebellum as a reference region due its low content of dopaminergic neurons^60,64^. For each subject, we measured the magnitude of dexamphetamine-induced dopamine release bilaterally in each striatal sub-region. Specifically, this was quantified as the reduction in BP_ND_ from the baseline condition (BP_ND_^Base^) to the post-dexamphetamine condition (BP_ND_^Amph^), expressed as a percentage of BP_ND_^Base^:

$$\Delta {\mathrm {BP}}_{{\mathrm {ND}}} = 100 \times \frac{{{\mathrm {BP}}_{{\mathrm {ND}}}^{{\mathrm {Base}}} - {\mathrm {BP}}_{{\mathrm {ND}}}^{{\mathrm {Amph}}}}}{{{\mathrm {BP}}_{{\mathrm {ND}}}^{{\mathrm {Base}}}}}{\mathrm{\% }}.$$

### Statistical analyses

Statistical Package for the Social Sciences (SPSS) version 24 was used for all statistical analysis (IBM, Armonk, N.Y.). A two-tailed significance alpha level of 0.05 was used for all tests. In order to examine potential confounds, we used Pearson’s correlation coefficient, independent *t*-test and analysis of covariance (ANCOVA) to investigate whether clinico-demographic factors reported to impact dopamine release, such as gender, nicotine, alcohol, cannabis morphine or stimulants use^49,65–70^, were also related to CTQ scores in our sample. Our primary hypothesis was that dexamphetamine-induced positive psychotic symptoms could be predicted by the interaction between childhood trauma and dexamphetamine-induced dopamine release, in accordance with previously described models^4,35,36,71^.

First, we tested whether childhood trauma or dopamine release capacity were correlated with dexamphetamine-induced symptoms using Pearson’s correlation coefficient. Next, we investigated whether the interaction between these two variables was a significant predictor of positive psychotic symptoms, by running a linear regression model, which included dexamphetamine-induced change in positive psychotic symptoms as the dependent variable, and the following predictor (independent) variables: ventral striatum dopamine release capacity (ΔBP_ND_), childhood trauma (CTQ score), and the interaction between these variables (ΔBP_ND_ × CTQ score). Our primary hypothesis was that the coefficient term (*β*) associated with the interaction term would be significantly greater than zero.

Second, we tested the specificity of the results by conducting additional multivariate regression models. To test whether our hypothesized interaction effect was specific for dopamine release (functional specificity), we repeated our models after substituting baseline D_2/3_R availability within the ventral striatum in the place of dopamine release. To test the anatomical specificity, we repeated the model after substituting in dexamphetamine-induced dopamine release within the associative or sensorimotor striatum, in place of the ventral striatum. Finally, to test the symptom domain specificity of the relationship, we repeated our models with the negative and general symptom sub-scale scores of the PANSS as the dependent variable (both induced symptom change and baseline symptoms).

Third, in order to examine whether the relationship between childhood trauma and positive psychotic symptoms is mediated by dopamine release, we performed a mediation analysis with childhood trauma as the independent variable, ventral striatal dexamphetamine-induced dopamine release as the mediator variable, and dexamphetamine-induced positive symptoms as the dependent variable, using the ‘Model 4’ template implemented in the PROCESS (version 3) SPSS package (http://www.processmacro.org/index.html)^72^. Effect sizes were estimated using 5000 bias corrected bootstrap samples. Mediation was deemed to be significant if 0 was not contained within the 95% bootstrap confidence intervals of the indirect effect, testing the combined effects of paths a (CTQ to dexamphetamine-induced dopamine release) and b (dexamphetamine-induced dopamine release to dexamphetamine-induced positive psychotic symptoms).

## Results

**Fig. 2:**
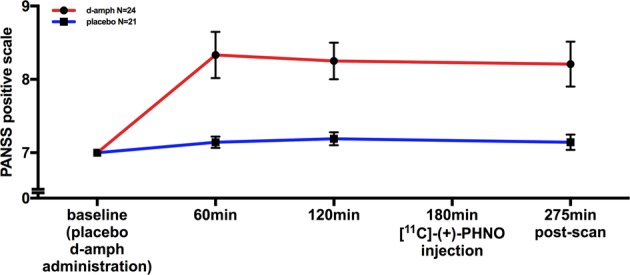
Scatter plot showing the mean (SEM) dexamphetamine-induced psychosis scores after placebo (blue line) and dexamphetamine (red line) administration (minimum score 7). Dexamphetamine administration significantly increased positive psychotic symptom scores compared to placebo (*t*(22) = 6.320, *p* < 0.001). d-amph dexamphetamine

Twenty-four participants underwent a baseline (placebo) [^11^C]-(+)-PHNO PET scan and a scan after oral dexamphetamine on a separate occasion (13 males, mean (SD) age = 23.4 (4.20) years). One male participant was excluded before data analysis due to a positive urine drug test for cannabis, as cannabis has been shown to influence D_2/3_ binding potential^68^. Participant demographics, PANSS positive scores and substance use are shown in Table 1. Participant scan parameters are shown in Table 2. Dexamphetamine administration resulted in a significant reduction in BP_ND_ relative to the baseline scan (*t*(23) = 14.29, *p* < 0.001), indicating significant dopamine release following dexamphetamine (Table 2), and significantly increased positive psychotic symptom scores (PANSS positive items) (*t*(22) = 6.32, *p* < 0.001) (Table 1 and Fig. 2). With a mean (SD) CTQ score of 36.7 (19.73) (range 25–97), our sample has values slightly higher than values generally reported in healthy adult samples (for example: mean (SD) of 31.4 (9.8)^73^ or 34.8 (9.73)^74^). However, they are lower than those reported in patients with psychosis (mean (SD) of 40.7 (11.4)^75^) or depression (mean (SD) of 40.5 (16.1)^73^ or 41.63 (10.23)^74^). The peak increase in PANSS positive was at 60 min post-dosing, with a mean (SD) increase of 1.44 (1.55). This is in line with increases reported in a previous study in healthy volunteers using amphetamine, which found a mean (SD) increase in PANSS of 1.6 (0.5) at 60 min post amphetamine dose^76^. We found no relationship between CTQ and age (Pearson’s correlation = 0.30, *p* = 0.152), and no differences between men and women’s CTQ scores (*t*(22) = −0.50, *p* = 0.621). All participants abstained from drugs, alcohol and nicotine on scan days, and an ANCOVA analysis showed no relationship between CTQ scores and current alcohol use (*F*(1,15) = 1.19, *p* = 0.294), past ecstasy (*F*(1,15) = 0.39, *p* = 0.543), cocaine (*F*(1,15) = 0.11, *p* = 0.741) and cannabis (*F*(2,15) = 0.37, *p* = 0.700) use. There were not enough participants who were smokers (*n* = 2) to make analysis of this a co-variable meaningful. No participants had a history of amphetamine use, heroin use or morphine use (see Table 1).

**Table 1:** Demographics, substance use and PANSS/childhood trauma scores

	Participants *N* = 24
Female	11
Male	13
Age	23.41 (4.20)
Tobacco smokers, (*n*)	2
Tobacco use (cigarettes/day), mean (SD)	
^a^Alcohol use (UK alcohol units/week), mean (SD)	4.83 (4.8)
Current cannabis	0
Past cannabis 0/1/2/3/4	15/4/4/1/0
Past cocaine use 0/1/2/3/4	22/1/1/0/0
Past amphetamine use 0/1/2/3/4	24/0/0/0/0
Past ecstasy use 0/1/2/3/4	19/5/0/0/0
Past morphine use 0/1/2/3/4	24/0/0/0/0
Past heroine use 0/1/2/3/4	24/0/0/0/0
Dexamphetamine PANSS positive score baseline, mean (SD)	7.00 (0)
Dexamphetamine PANSS positive score 60 min, mean (SD)	8.33 (1.55)
Dexamphetamine PANSS positive score 120 min, mean (SD)	8.25 (1.22)
Dexamphetamine PANSS positive score 275 min, mean (SD)	8.21 (1.50)
Placebo PANSS positive score baseline, mean (SD)	7.00 (0)
Placebo PANSS positive score 60 min, mean (SD)	7.14 (0.36)
Placebo PANSS positive score 120 min, mean (SD)	7.19 (0.40)
Placebo PANSS positive score 275 min, mean (SD)	7.14 (.48)
CTQ, mean (SD)	36.7 (19.73)

For drug use, categories of 0/1/2/3/4/5 indicate never used/very occasional or experimental use/occasional or monthly use/moderate or weekly use/severe or daily use, respectively

*CTQ* Childhood Trauma Questionnaire, *d-amph* dexamphetamine, *D*_*2/3*_*R* dopamine D_2/3_ receptor

^a^UK alcohol unit = 10 ml ≅ 7.88 g alcohol

**Table 2:** Scanning data

	Participants *N* = 24
Injected radioactivity baseline (MBq), mean (SD)	183.04 (45.91)
Injected mass baseline (μg), mean (SD)	1.63 (0.32)
Injected radioactivity d-amph (MBq), mean (SD)	159.89 (53.22)
Injected mass d-amph (μg), mean (SD)	1.60 (0.37)
% displacement ventral striatum, mean (SD)	21.79 (6.26)
% displacement associative striatum, mean (SD)	12.90 (5.47)
% displacement sensorimotor striatum, mean (SD)	21.38 (5.49)
D_2/3_R availability ventral striatum, mean (SD)	2.82 (0.34)
D_2/3_R availability associative striatum, mean (SD)	1.95 (0.29)
D_2/3_R availability sensory motor striatum, mean (SD)	2.02 (0.23)

*CTQ* Childhood Trauma Questionnaire, *d-amph* dexamphetamine, *D*_*2/3*_*R* dopamine D_2/3_ receptor, *MBq* megabecquerel

### Bivariate relationship between– childhood trauma or dopamine release capacity and symptoms

Our primary hypothesis was that the interaction between childhood trauma and dopamine release capacity would be a significant predictor of dexamphetamine-induced positive psychotic symptoms. We first tested the simple bivariate relationships between childhood trauma or ventral striatum dopamine release and dexamphetamine-induced positive psychotic symptoms. Pearson’s correlations showed that there was no significant correlation between childhood trauma and dexamphetamine-induced positive psychotic symptoms (*r* = 0.348, *p* = 0.096, *n* = 24), nor between ventral striatum dopamine release capacity and dexamphetamine-induced positive psychotic symptoms (*r* = 0.111, *p* = 0.606, *n* = 24). Unexpectedly, we found a negative correlation between childhood trauma load and ventral striatal dopamine release (Pearson’s *r* = −0.488, *p* = 0.016) (Supplementary Fig. 1). Having established that neither childhood trauma nor dopamine release capacity in isolation were significant predictors of positive psychotic symptoms in healthy individuals, we tested the predictive significance of the interaction between these two variables using a linear regression model (Table 3).

**Table 3:** General linear model 1

GLM1 Increase in PANSS positive ~ β0 + β1*ΔBP_ND_ + β2*CTQ + β3*[ΔBP_ND_*CTQ]					
*F*		Adjusted *R*^2^	*p* Value vs. constant model		
*F*(20,3) = 6915		0.44	0.002		
	Unstandardized coefficients		Standardized coefficients	*t*	*p* Value
	*β*	Std. error	*β*		
Constant (*β*0)	2.22	1.326		1.674	0.11
ΔBP_ND_ (*β*1)	−0.153	0.07	−0.914	−2.191	0.04
CTQ (*β*2)	−0.077	0.032	−1.441	−2.38	0.027
ΔBP_ND_ × CTQ (*β*3)	0.007	0.002	1.828	3.405	0.003

*ΔBP*_*ND*_ ventral striatum dopamine release capacity (%), *CTQ* Childhood Trauma Questionnaire, *GLM* general linear model

The general linear model including dexamphetamine-induced positive psychotic symptoms as dependent variable was superior to the null-model in predicting symptom scores and providing evidence that the interaction between childhood trauma and mesolimbic dopamine sensitivity (interaction term: ΔBP_ND_ × CTQ score) was a predictor of dexamphetamine-induced positive psychotic symptoms

**Fig. 3:**
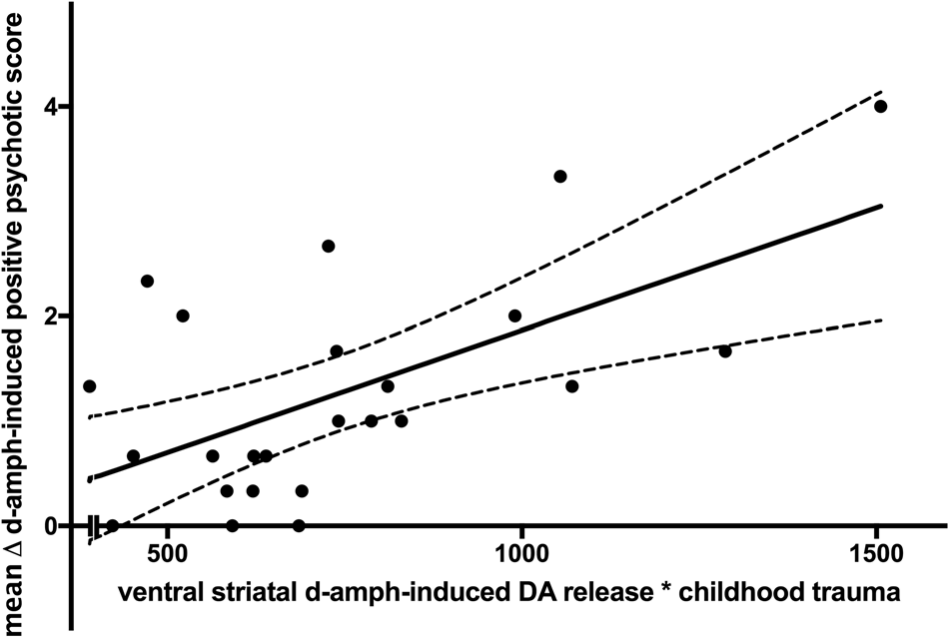
Scatter plot showing the interaction between dopamine release and childhood trauma exposure in predicting the induction of psychotic symptoms by dexamphetamine. The interaction term significantly predicts dexamphetamine-induced positive psychotic symptoms (*p* = 0.003), indicating that dopamine release and childhood trauma load interact to increase the induction of positive psychotic symptoms following dexamphetamine. DA dopamine, d-amph dexamphetamine

The general linear model (GLM1) including dexamphetamine-induced positive psychotic symptoms as the dependent (outcome) variable (ΔPANSS, the mean increase in PANSS score over the three post-amphetamine time-points), with three independent (predictor) variables (ventral striatum dopamine release capacity, childhood trauma load, and the interaction between these two variables) was superior to the null-model in predicting symptom scores (Table 2, *F*(20,3) = 6.915, *p*-value vs. constant model = 0.002). Importantly, the regression coefficient associated with the interaction term in this model was significantly greater than zero, indicating that the interaction between childhood trauma and dexamphetamine-induced dopamine release predicted dexamphetamine-induced positive psychotic symptoms (*p* = 0.003; Table 2 and Fig. 3). Calculating dexamphetamine-induced positive psychotic symptom severity as the *peak* post-dosing PANSS-positive score did not change the nature of the results (Supplementary Table 1, GLM2, *p*-value vs. constant model = 0.004, interaction term (ΔBP_ND_ × CTQ*) p* = 0.004), nor did restricting the analysis of symptom induction to the combined ‘delusions’, ‘hallucinations’ and ‘suspiciousness’ items of the PANSS (Supplementary Table 1, GLM3, *p*-value vs. constant model = 0.011, interaction term (ΔBP_ND_ × CTQ) *p* = 0.023).

### Mediation analysis

**Fig. 4:**
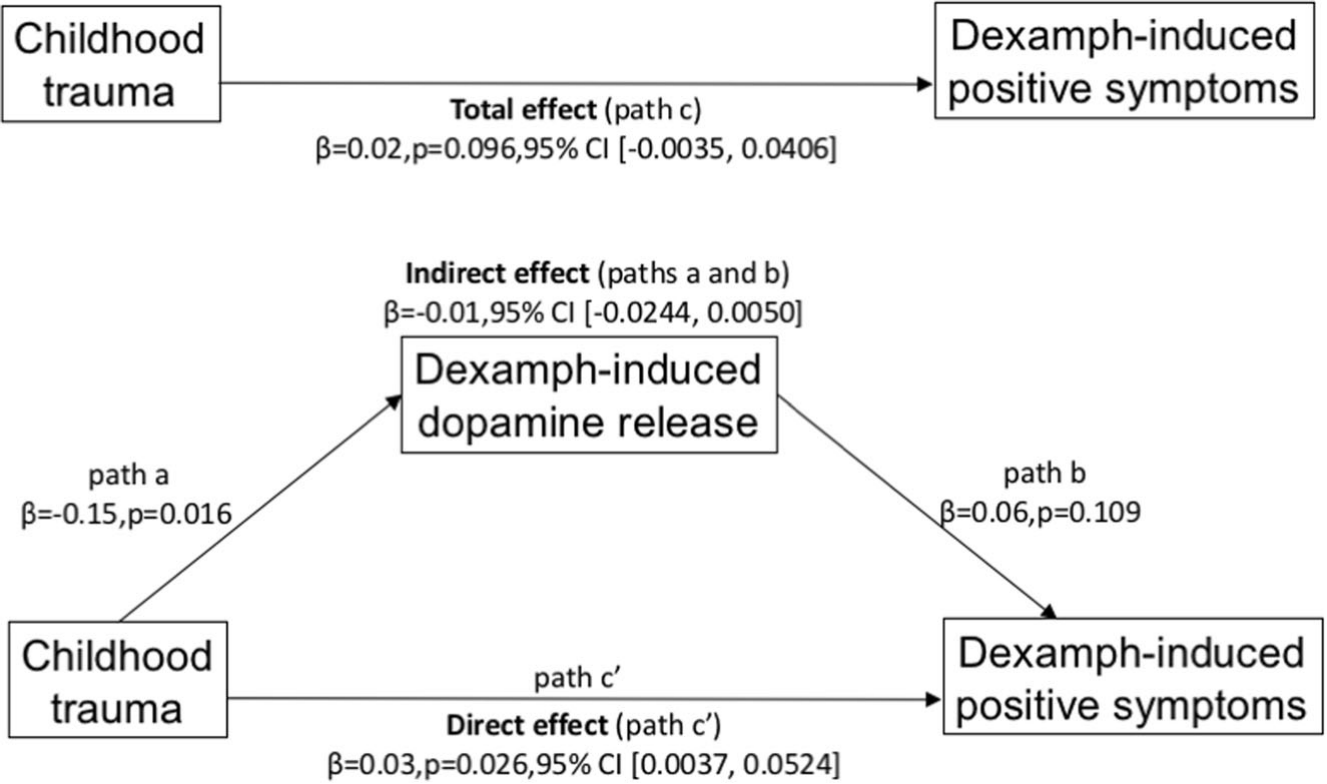
Mediation analysis between childhood trauma load, ventral striatal dopamine release and -induced positive symptoms. There was no significant mediation effect of childhood trauma on dexamphetamine-induced positive symptoms through dexamphetamine-induced dopamine release *β* = −0.01 (standardized *β* = −0.18), 95% CI (−0.0244, 0.0050), *p* = 0.158 (Sobel test)

The mediation analysis testing whether dopamine release mediates childhood trauma in predicting positive symptoms is shown in Fig. 4. The mediation analysis was not significant (*β* = −0.01, 95% CI (−0.0244, 0.0050), Sobel test’s *p* = 0.158). This indicates that there was no significant indirect effect of childhood trauma on dexamphetamine-induced positive symptoms (i.e. an effect mediated through dexamphetamine-induced dopamine release).

### Specificity of the relationship between ventral striatum dopamine release capacity and positive psychotic symptoms

Having shown that the interaction between ventral striatum dopamine release and childhood trauma is a significant predictor of dexamphetamine-induced positive symptoms, we tested the functional and anatomical specificity of this relationship within the dopamine system, and its specificity for positive psychotic symptoms. Functionally, the relationship between ventral striatum dopamine function and induced positive psychotic symptoms was specific to dopamine release capacity and was not significant for ventral striatum baseline D_2/3_R availability (Supplementary Table 1, GLM4). Anatomically, the significant interaction between dopamine release capacity and childhood trauma in predicting induced positive psychotic symptoms was specific to the ventral striatum, and was not significant when release capacity was measured in the associative and sensorimotor striatal subdivisions (Supplementary Table 1, GLM5). Finally, we investigated whether the association between childhood trauma and ventral striatum dopamine release capacity was specific to positive symptoms. Further regression analyses confirmed that neither childhood trauma or ventral striatal dopamine release nor their interaction, was a significant predictor of dexamphetamine-induced change in negative or general PANSS subscales, and they were also not predictors of baseline negative and PANSS general subscale (a proxy for baseline non-psychotic psychopathology) (Supplementary Table 1, GLM7–10).

## Discussion

Our results indicate that childhood trauma influences the relationship between ventral striatal dopamine release and induced positive psychotic symptoms, with a significant moderation but not mediation effect (Figs. 2, 3). This supports the hypothesis that childhood trauma interacts with the dopaminergic response to amphetamine to increase the induction of positive psychotic symptoms (moderation effect), but not that childhood trauma leads to sensitization of dopamine release (mediation effect). This extends previous findings by showing for the first time the link between childhood trauma, dopamine release and induced positive psychotic symptoms.

We did not find significant relationships between positive psychotic symptoms or childhood trauma and baseline D_2/3_R availability, consistent with evidence there are no marked in vivo D_2/3_R alterations in schizophrenia^77,78^. Moreover, there was no relationship with dopamine release in the associative and sensorimotor striatal subdivisions or with negative and general symptoms, indicating the effect is on the ventral striatum and positive psychotic symptoms. This is consistent with prior literature implicating dopaminergic changes in the ventral striatum following exposure to environmental and early life stressors, and the role of the ventral striatum in limbic cortico-striatal circuits relevant to processing of affective and reward stimuli^43,44,46^.

### Interaction between childhood trauma and ventral striatal dopamine release

The interaction between childhood trauma load and ventral striatal dopamine release is in line with the large body of literature showing the role of the ventral striatum in stimulant-induced dopamine release after exposure to environmental stressors. However, we did not find any positive relationship between history of childhood trauma and induced striatal dopamine release—instead the relationship in our sample was negative (Pearson’s *r* = −0.488, *p* = 0.016) (Supplementary Fig. 1). This is seemingly at odds with predictions from theories proposing that childhood trauma sensitizes the dopamine system to psychological and pharmacological challenge in later life^4,35^. Rodents exposed to early stressors exhibit increased dopamine levels after stimulant administration in the nucleus accumbens (ventral striatum)^39–42,79,80^. Human PET studies have also shown increased stress-induced dopamine release in the ventral striatum in people with history of low maternal care^43^, as well as increased amphetamine-induced dopamine-release in individuals with a greater number of traumatic events in childhood^44^ and individuals with hearing impairment^47^. Furthermore, positive correlations between amphetamine-induced dopamine release in the ventral striatum and plasma cortisol levels have also been observed in healthy individuals^46,81^. Oswald et al. found that the relationship between childhood traumatic exposure and dopamine release was mediated by perceived stress in adulthood, where higher levels of perceived stress were associated with higher ventral striatal dopamine responses to amphetamine^44^. This observation may explain the apparent inconsistency between our findings and these previous findings if there is lower perceived stress in adulthood in people with high CTQ scores in our sample. However, as we did no measure perceived stress in adulthood, this remains to be tested and highlights that it would be useful to measure perceived stress as well in future studies. Nevertheless, the interaction between childhood trauma load and ventral striatal dopamine release on the induction of psychotic symptoms indicates that a history of greater childhood trauma increases an individual’s vulnerability to the psychotogenic effects of striatal dopamine release, in line with cognitive schema-interpretation model of psychosis^4^.

### The potential mechanism underlying the interaction effect on the induction of psychotic symptoms

Human fMRI studies implicate the ventral striatum in errors of salience attribution for both appetitive, aversive stimuli^82–85^, and specifically indicates its role in belief updating following salient stimuli^86^. Ventral striatal dopamine regulates the processing of the salience of stimuli, and, by increasing dopamine release, amphetamine is thought to disrupt this to lead to salience being aberrantly assigned to stimuli^8,87,88^. Externalizing cognitive schema, such as being more likely to interpret experiences to be threatening or out of personal control are associated with a greater likelihood of psychotic symptoms in clinical and non-clinical samples^8,88–91^. Childhood trauma is characterized by experiences of threat and loss of control to others, which could lead to more negative or externalizing cognitive schema. Supporting this, childhood trauma has been associated with hostile attribution biases and negative self-schema^92,93^. Thus, this evidence suggests the interaction we observe between childhood trauma and dopamine release in the induction of psychotic symptoms could be explained by childhood trauma altering cognitive schema to be more externalizing, leading to a greater likelihood of a psychotic-like interpretation of aberrant salience following dopamine release by amphetamine. Whilst this interpretation is speculative, this could be tested in future studies by determining if cognitive schema mediate the interaction between traumatic childhood experiences and dopamine release on the induction of psychotic symptoms. It should also be recognized that other brain regions, in particular frontal cortical regions, are involved in salience processing and altered cortico-striatal connectivity has been found in people with a history of childhood trauma^94^ and linked to striatal dopamine release^95^, indicating other brain regions could also be involved in mediating this interaction.

### Specificity of the findings to the ventral (limbic) striatum

Our finding that the effect is specific to the limbic striatum contrasts with those of a recent meta-analysis indicating that the main locus of increased dopamine synthesis capacity in schizophrenia is the associative striatum, and not the ventral (limbic) striatum^96^. However, it is not known whether these meta-analytic findings can be extended to pharmacologically induced dopamine release as there are conflicting results in the literature. One study in patients with schizophrenia and comorbid substance dependence showed significant associations between amphetamine-induced dopamine release in the ventral striatum and the precommissural caudate (associative striatum) and change in positive symptoms^97^, whilst another study using [^18^C]-fallypride found an association between schizotypal traits and amphetamine-induced dopamine release in the head of the caudate but extending into the ventral striatum^98^. Two other studies found that patients with schizophrenia had a similar range of BP_ND_ reduction in the ventral striatum and sensorimotor striatum at 6–10 h following dexamphetamine administration^99^, and that patients with schizophrenia had mean (SD) percentage reduction of 17.6% (7.8) in the ventral striatum and 24.4% (7.2) in the sensorimotor striatum^22^. To our knowledge, none of the other studies of amphetamine-induced dopamine release have examined sub-regional differences in striatal dopamine release in schizophrenia^16–21^. The ventral striatum is the most sensitive region to amphetamine-induced dopamine release^56,59,100–102^. Interestingly, a study examining amphetamine sensitization in healthy individuals found that an initial dose of amphetamine caused dopamine release in the ventral striatum whilst further doses at 14 and 365 days elicited increased dopamine release in the ventral striatum relative to the initial dose, with sensitized dopamine release progressively extending to the dorsal caudate and putamen^103^. Furthermore, a longitudinal study of people scanned in the prodrome and then again when acutely psychotic showed progressive increases in dopamine synthesis capacity in the dorsal striatum^104^. Thus, these findings could suggest that the initial dopaminergic response is in the ventral striatum which then extends to the dorsal striatum with the development of a psychotic disorder, but further studies are needed to confirm this hypothesis. Lastly, the regional specificity of the findings to the ventral striatum might also reflect tracer-specific factors. [^11^C]-(+)-PHNO is more sensitive to amphetamine-induced dopamine release in the ventral striatum than D_2/3_R antagonist tracers such as [^11^C]-raclopride^56^, a property that may be related to its high affinity for the D_3_R, which is more abundant in the ventral compared to the associative striatum and [^11^C]-(+)-PHNO.

## Limitations

The first limitation of this study is that all individuals were healthy volunteers, limiting the generalizability of these findings to patients with psychotic disorders. This may account for the relatively small increase in PANSS score following dexamphetamine. Further studies are therefore needed to examine the relationships between childhood trauma, dopamine release and positive psychotic symptoms in clinical populations, and to examine stress-induced dopamine release. Second, the study was cross-sectional and exposure to childhood trauma was assessed retrospectively via questionnaires, and could be influenced by recall bias. However, retrospective assessment of childhood trauma has been shown to be reliable in both healthy population and patients with psychosis^105^. Lastly, it is possible that a third variable that correlates with both dopamine release and CTQ mediates the relationship we observe. However, we found no evidence that clinico-demographic factors that have been reported to be related to dopamine release such as gender, nicotine, alcohol, cannabis morphine or stimulants^49,65–70^ are related to CTQ scores in our sample.

## Conclusions

Childhood trauma and ventral striatal dopamine release interact to influence the emergence of positive psychotic symptoms following dexamphetamine. This is not consistent with a simple dopamine sensitization model for the link between childhood trauma and psychosis risk, but could, instead, suggest that childhood trauma moderates the cognitive response to dopamine release to make psychotic experiences more likely.

## Supplementary information

Supplementary information

Suppl.Figure1
