# Supplementary material for: The relationship between childhood trauma, dopamine release and dexamphetamine-induced positive psychotic symptoms: a [11C]-(+)-PHNO PET study
Source: Transl Psychiatry. 2019 Nov 11;9:287. doi: 10.1038/s41398-019-0627-y (PMC6848217; doi:10.1038/s41398-019-0627-y)
Supplement: Supplementary file 1 — Supplementary information [file 41398_2019_627_MOESM1_ESM.docx]

# Supplementary information

Supplementary Figure 1

Scatter plot showing a negative correlation between childhood trauma load and dexamphetamine-induced dopamine release (Pearson r=-0.488, p=0.016).

Supplementary Table 1: Supplementary General Linear Models

| **GLM2 Dexamphetamine-induced change in positive psychotic symptoms (peak values)** | | | | | | |
| --- | --- | --- | --- | --- | --- | --- |
| *Increase in PANSS positive (peak values) ~ β0 + β1*ΔBP_ND_ + β2*CTQ + β3*[ΔBP_ND_*CTQ]* | | | | | | |
| F | | Adjusted R-Squared | | p value vs. constant model | | |
| F(20,3)=5.975 | | 0.394 | | 0.004 | | |
|  | Unstandardized Coefficients | |  | Standardized Coefficients | t | p value |
|  | β | | Std. Error | β |  |  |
| *constant* (β0) | 3.915 | | 2.139 |  | 1.83 | 0.082 |
| ventral striatum *ΔBP_ND_ (β1)* | -0.244 | | 0.113 | -0.936 | -2.166 | 0.043 |
| *CTQ (β2*) | -0.121 | | 0.052 | -1.467 | -2.338 | 0.03 |
| *ΔBP_ND_*CTQ (β3)* | 0.011 | | 0.003 | 1.816 | 3.263 | 0.004 |
| **GLM3 Dexamphetamine-induced change in positive psychotic symptoms (Delusions, Hallucinatory Behaviour and Suspiciousness items)** | | | | | | |
| *Increase in PANSS positive (peak values) ~ β0 + β1*ΔBP_ND_ + β2*CTQ + β3*[ΔBP_ND_*CTQ]* | | | | | | |
| F | | Adjusted R-Squared | | p value vs. constant model | | |
| F(20,3)=4.810 | | 0.332 | | 0.011 | | |
|  | Unstandardized Coefficients | |  | Standardized Coefficients | t | p value |
|  | β | | Std. Error | β |  |  |
| *constant* (β0) | 0.292 | | 1.101 |  | 0.266 | 0.793 |
| ventral striatum *ΔBP_ND_ (β1)* | -0.067 | | 0.058 | -0.521 | -1.149 | 0.264 |
| *CTQ (β2*) | -0.043 | | 0.027 | -1.063 | -1.614 | 0.122 |
| *ΔBP_ND_*CTQ (β3)* | 0.004 | | 0.002 | 1.438 | 2.463 | 0.023 |
| **Functional specificity**  **GLM4 Baseline ventral striatal D**2^high^**/3R binding potential and dexamphetamine-induced change in positive psychotic symptoms** | | | | | | |
| *Increase in PANSS positive ~ β0 + β1*BP_ND_ + β2*CTQ + β3*[BP_ND_*CTQ]* | | | | | | |
| F | | Adjusted R-Squared | | p value vs. constant model | | |
| F(20,3)=1.110 | | 0.014 | | 0.368 | | |
|  | Unstandardized Coefficients | |  | Standardized Coefficients | t | p value |
|  | β | | Std. Error | β |  |  |
| *constant* (β0) | -1.248 | | 3.614 |  | -0.345 | 0.733 |
| ventral striatum *BP_ND_ (β1)* | 0.658 | | 1.252 | 0.212 | 0.525 | 0.605 |
| *CTQ (β2*) | 0.033 | | 0.076 | 0.611 | 0.429 | 0.673 |
| *BP_ND_*CTQ (β3)* | -0.005 | | 0.025 | -0.304 | -0.201 | 0.843 |
| **Anatomical specificity**  **GLM5 Associative striatal dopamine release and dexamphetamine-induced change in positive psychotic symptoms** | | | | | | |
| *Increase in PANSS positive ~ β0 + β1*ΔBP_ND_ + β2*CTQ + β3*[ΔBP_ND_*CTQ]* | | | | | | |
| F | | Adjusted R-Squared | | p value vs. constant model | | |
| F(20,3)=0.999 | | 0.000 | | 0.414 | | |
|  | Unstandardized Coefficients | |  | Standardized Coefficients | t | p value |
|  | β | | Std. Error | β |  |  |
| *constant* (β0) | 0.393 | | 1.246 |  | 0.315 | 0.756 |
| associative striatum *ΔBP_ND_ (β1)* | 0.012 | | 0.091 | 0.062 | 0.131 | 0.897 |
| *CTQ (β2*) | 0.017 | | 0.031 | 0.322 | 0.546 | 0.591 |
| *ΔBP_ND_*CTQ (β3)* | 0.000 | | 0.002 | 0.052 | 0.079 | 0.938 |
| **Anatomical specificity**  **GLM6 Sensorimotor striatal dopamine release and dexamphetamine-induced change in positive psychotic symptoms** | | | | | | |
| *Increase in PANSS positive ~ β0 + β1* ΔBP_ND_ + β2*CTQ + β3*[ ΔBP_ND_ *CTQ]* | | | | | | |
| F | | Adjusted R-Squared | | p value vs. constant model | | |
| F(20,3)=1.250 | | 0.032 | | 0.318 | | |
|  | Unstandardized Coefficients | |  | Standardized Coefficients | t | p value |
|  | β | | Std. Error | β |  |  |
| *constant* (β0) | -0.835 | | 2.201 |  | -0.379 | 0.708 |
| sensorimotor striatum *ΔBP_ND_ (β1)* | 0.066 | | 0.105 | 0.343 | 0.623 | 0.540 |
| *CTQ (β2*) | 0.038 | | 0.059 | 0.718 | 0.652 | 0.522 |
| *ΔBP_ND_*CTQ (β3)* | -0.001 | | 0.003 | -0.356 | -0.314 | 0.757 |
| **Symptom specificity**  **GLM7 Dexamphetamine-induced change in PANSS negative subscale** | | | | | | |
| *Increase in PANSS negative ~ β0 + β1* ΔBP_ND_ + β2*CTQ + β3*[ΔBP_ND_*CTQ]* | | | | | | |
| F | | Adjusted R-Squared | | p value vs. constant model | | |
| F(20,3)=1.372 | | 0.046 | | 0.280 | | |
|  | Unstandardized Coefficients | |  | Standardized Coefficients | t | p value |
|  | β | | Std. Error | β |  |  |
| *constant* (β0) | 0.283 | | 0.752 |  | 0.377 | 0.710 |
| ventral striatum *ΔBP_ND_ (β1)* | -0.004 | | 0.04 | -0.055 | -0.101 | 0.920 |
| *CTQ (β2*) | 0.01 | | 0.018 | 0.424 | 0.540 | 0.595 |
| *ΔBP_ND_*CTQ (β3)* | -0.001 | | 0.001 | -0.539 | -0.773 | 0.449 |
| **Symptom specificity**  **GLM8 Baseline PANSS negative subscale** | | | | | | |
| *Baseline PANSS negative ~ β0 + β1* ΔBP_ND_ + β2*CTQ + β3*[ ΔBP_ND_*CTQ]* | | | | | | |
| F | | Adjusted R-Squared | | p value vs. constant model | | |
| F(20,3)=1.223 | | 0.028 | | 0.327 | | |
|  | Unstandardized Coefficients | |  | Standardized Coefficients | t | p value |
|  | β | | Std. Error | β |  |  |
| *constant* (β0) | 6.836 | | 0.744 |  | 9.193 | 0 |
| ventral striatum *ΔBP_ND_ (β1)* | 0.000 | | 0.039 | -0.005 | -0.010 | 0.992 |
| *CTQ (β2*) | -0.011 | | 0.018 | -0.488 | -0.615 | 0.546 |
| *ΔBP_ND_*CTQ (β3)* | 0.001 | | 0.001 | 0.580 | 0.824 | 0.42 |
| **Symptom specificity**  **GLM9 Dexamphetamine-induced change in PANSS general subscale** | | | | | | |
| *Increase in PANSS general ~ β0 + β1* ΔBP_ND_ + β2*CTQ + β3*[ ΔBP_ND_ *CTQ]* | | | | | | |
| F | | Adjusted R-Squared | | p value vs. constant model | | |
| F(20,3)=2.886 | | 0.197 | | 0.061 | | |
|  | Unstandardized Coefficients | |  | Standardized Coefficients | t | p value |
|  | β | | Std. Error | β |  |  |
| *constant* (β0) | -1.914 | | 3.576 |  | -0.535 | 0.598 |
| ventral striatum *ΔBP_ND_ (β1)* | -0.027 | | 0.189 | -0.070 | -0.142 | 0.889 |
| *CTQ (β2*) | 0.025 | | 0.087 | 0.208 | 0.288 | 0.777 |
| *ΔBP_ND_*CTQ (β3)* | 0.003 | | 0.006 | 0.369 | 0.577 | 0.571 |
| **Symptom specificity**  **GLM10 Baseline PANSS general subscale** | | | | | | |
| *Baseline PANSS general ~ β0 + β1*ΔBP_ND_ + β2*CTQ + β3*[ ΔBP_ND_ *CTQ]* | | | | | | |
| F | | Adjusted R-Squared | | p value vs. constant model | | |
| F(20,3)=0.345 | | -0.093 | | 0.793 | | |
|  | Unstandardized Coefficients | |  | Standardized Coefficients | t | p value |
|  | β | | Std. Error | β |  |  |
| *constant* (β0) | 17.565 | | 2.540 |  | 6.915 | 0.000 |
| ventral striatum *ΔBP_ND_ (β1)* | -0.014 | | 0.134 | -0.059 | -0.102 | 0.920 |
| *CTQ (β2*) | -0.004 | | 0.062 | -0.060 | -0.072 | 0.944 |
| *ΔBP_ND_*CTQ (β3)* | -0.001 | | 0.004 | -0.166 | -0.222 | 0.827 |
| *ΔBP_ND_*=dopamine release capacity (%)  *CTQ*=Childhood Trauma Questionnaire  GLM=General Linear Model | | | | | | |
