# Supplementary figures and images for: The relationship between childhood trauma, dopamine release and dexamphetamine-induced positive psychotic symptoms: a [11C]-(+)-PHNO PET study
Source: Transl Psychiatry. 2019 Nov 11;9:287. doi: 10.1038/s41398-019-0627-y (PMC6848217; doi:10.1038/s41398-019-0627-y)

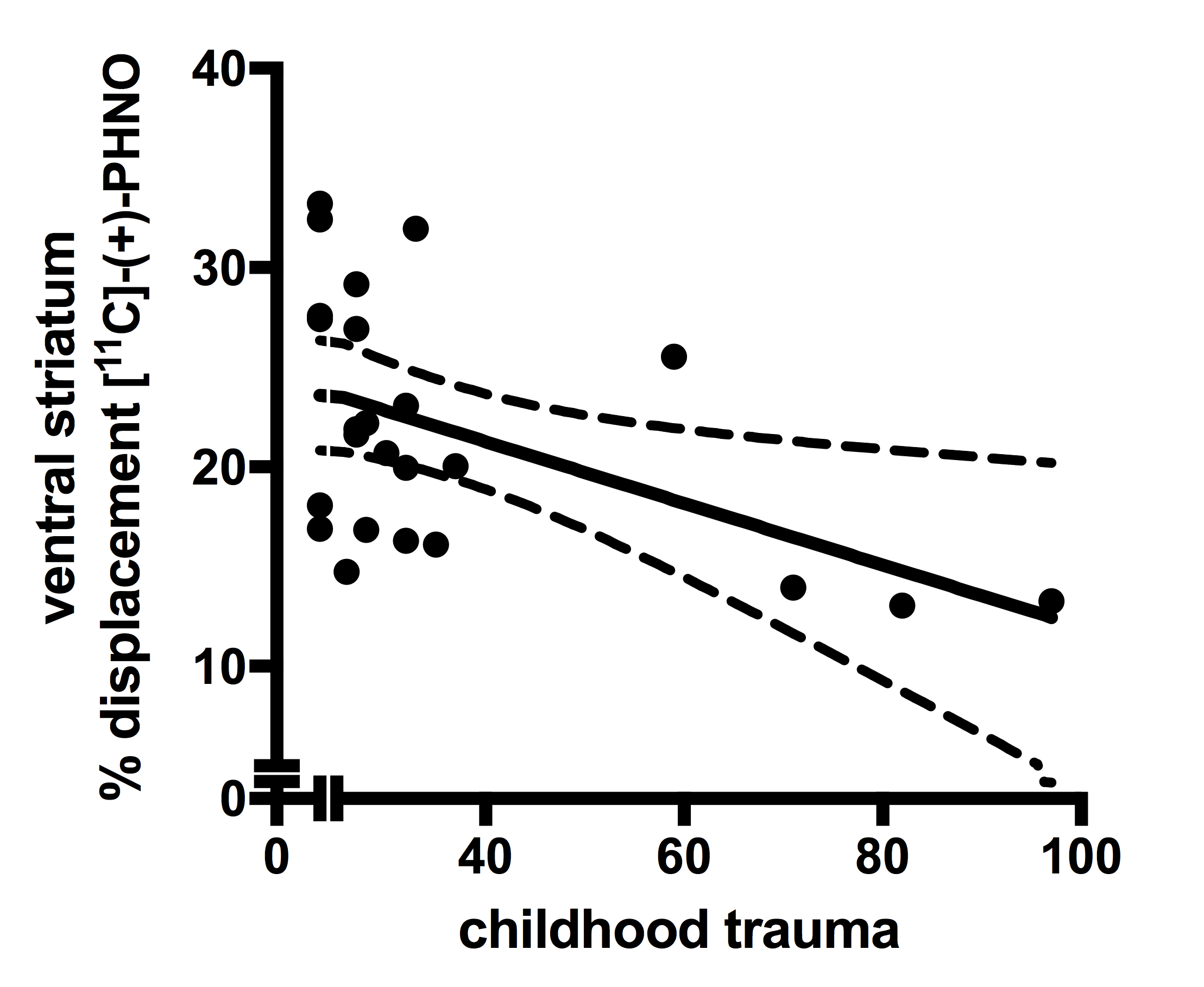

Supplement: Supplementary file 2 — Suppl.Figure1 [file 41398_2019_627_MOESM2_ESM.tif]
